# Serial soluble neurofilament heavy chain in plasma as a marker of brain injury after cardiac arrest

**DOI:** 10.1186/cc11244

**Published:** 2012-03-12

**Authors:** Malin Rundgren, Hans Friberg, Tobias Cronberg, Bertil Romner, Axel Petzold

**Affiliations:** 1 Department of Intensive-and Perioperative Care, Skåne University Hospital; Department of Clinical Sciences Lund University, Lund, Sweden; 2 Department of Neurology, Skåne University Hospital; Department of Clinical Sciences, Lund University, Lund, Sweden; 3 Department of Neurosurgery, Copenhagen University, Copenhagen, Denmark; 4 Department of Neuroimmunology, UCL Institute of Neurology, Queen Square, London, UK; 5 Deptartment of Neurology, VU medisch centrum Amsterdam, Amsterdam, NL

## Abstract

**Introduction:**

Induced hypothermia has been shown to improve outcome after cardiac arrest, but early prognostication is hampered by the need for sedation. Here we tested whether a biomarker for neurodegeneration, the neurofilament heavy chain (NfH), may improve diagnostic accuracy in the first days after cardiac arrest.

**Methods:**

This prospective study included 90 consecutive patients treated with hypothermia after cardiac arrest. Plasma levels of phosphorylated NfH (SMI35) were quantified using standard ELISA over a period of 72 h after cardiac arrest. The primary outcome was the dichotomized Cerebral Performance Categories scale (CPC). A best CPC 1-2 during 6 months follow-up was considered a good outcome, a best CPC of 3-4 a poor outcome. Receiver operator characteristics and area under the curve were calculated.

**Results:**

The median age of the patients was 65 years, and 63 (70%) were male. A cardiac aetiology was identified in 62 cases (69%). 77 patients (86%) had out-of-hospital cardiac arrest. The outcome was good in 48 and poor in 42 patients. Plasma NfH levels were significantly higher 2 and 36 hours after cardiac arrest in patients with poor outcome (median 0.28 ng/mL and 0.5 ng/mL, respectively) compared to those with good outcome (0 ng/mL, p = 0.016, p < 0.005, respectively). The respective AUC were 0.72 and 0.71.

**Conclusions:**

Plasma NfH levels correlate to neurological prognosis following cardiac arrest. In this study, 15 patients had neurological co-morbidities and there was a considerable overlap of data. As such, neurofilament should not be used for routine neuroprognostication until more data are available.

## Introduction

Post cardiac arrest intensive care is complex and the dynamic multi-organ failure is often referred to as the post-resuscitation syndrome [1]. The major cause of death in patients with return of spontaneous circulation (ROSC) is ischemic brain damage [2], which evolves over several days [3]. In many patients who remain comatose after cardiac arrest, a reliable assessment of neurological prognosis therefore needs to be postponed by several days [4-6]. The use of induced hypothermia to improve outcomes has further complicated matters because it mandates sedation and intermittent use of muscle relaxants during the intervention. Moreover, hypothermia delays the metabolism of drugs [7] and makes a clinical neurological examination less reliable [8,9]. Therefore, we need to reassess and improve our prognostic instruments and explore novel and complementary methods of a clinical neurological examination [10].

Several biomarkers have been evaluated to assess brain damage after cardiac arrest with inconsistent results - for example, neuronspecific enolase (NSE), S-100B, and glial fibrillary acidic protein (GFAP) [5,11-13]. Among these, NSE and S-100B are the most extensively studied and NSE is the only one that has been integrated into clinical guidelines [6]. Although highly correlated to the extent of ischemic brain injury [10], weaknesses with NSE include its sensitivity to false positives due to sample hemolysis and the lack of a standard [14,15]. The advantage of a high sensitivity of blood S-100B levels for parenchymal brain damage is hampered by a lower specificity due to presence in extra-nervous system tissues such as bone marrow and fatty tissue [16,17].

The neurofilament heavy chain (NfH) is a specific protein biomarker of neurons and axons [18]. This 190 to 210 kDa protein of various degrees of phosphorylation is an obligate heteropolymer with the Nf light (NfL) and medium (NfM) chains. Together NfH, NfL and NfM belong to the class IV group of intermediate filaments, which support the axonal cytoskeleton [18]. Accumulation or increased levels of NfH and NfL were observed in a number of diseases including stroke and cardiac arrest [18-24].

To investigate the time-course of NfH release and its prognostic value, we studied serial plasma samples in a cohort of hypothermia-treated cardiac arrest patients.

## Materials and methods

This prospective study was performed in the general and cardio-thoracic ICUs of Lund University Hospital, Sweden, from August 2003 to March 2007. The study was approved by the Regional Ethical Review Board at Lund University (411/2004, 223/2008), and informed consent was sought from next of kin or, retrospectively, from the patient.

Epidemiological data and cardiac arrest data were collected prospectively. All cardiac arrest patients, regardless of location of arrest or initial rhythm, with ROSC and sustained unconsciousness (Glasgow coma scale (GCS) ≤7), were considered for induced hypothermia. Exclusion criteria for hypothermia treatment were terminal disease, intracerebral hemorrhage, aortic dissection, or major trauma. Hypothermia was initiated as soon as possible after ROSC in the emergency room or catheterization laboratory using 30 ml/kg cold saline and subsequent treatment in the ICU was performed as described earlier [25].

A cardiologist initially evaluated all patients. Urgent angiography, percutaneous cardiac intervention and, if necessary, circulatory support using intraaortic balloon pump counter pulsations was undertaken when indicated. Patients received hypothermia for 24 hours at 33+/-1°C and rewarming was controlled at 0.5°C/hour. Patients were sedated using propofol 2 to 4 mg/kg/hour and fentanyl 1 to 3 μg/kg/hour [25].

In patients remaining comatose, full intensive care was provided for at least three days after normothermia, at which time a clinical neurological evaluation was performed. In addition, somatosensory evoked potentials (SSEP), amplitude-integrated electroencephalogram (aEEG) and diffusion-weighted magnetic resonance imaging (DW-MRI) were added as a basis for a decision on level of care [10]. The patients were evaluated at ICU and hospital discharge by an intensivist, and by a neurologist six months later, using the five-graded Cerebral Performance Categories (CPC) scale: CPC 1 = good cerebral performance, CPC 2 = moderate cerebral disability, independent, CPC 3 = severe cerebral disability, conscious but dependent, CPC 4 = coma, CPC 5 = death [26]. To assess for clinically relevant neurological injury, the best CPC score during six months follow up was regarded as the primary outcome. A best CPC score of 1 to 2 at any time was considered a good outcome and a best CPC of 3 to 4 a poor outcome. Our secondary outcome was survival at six months.

As the release profile of soluble NfH after cardiac arrest was not known, we chose to collect several samples over the first three days to assess the release profile in a similar fashion as previously done for neuron-specific enolase and S-100B [12], thus in part using a cohort on which we have previously published. Plasma-samples for soluble neurofilament analysis were collected at admission and at 2, 6, 12, 24, 36, 48, and 72 hours after cardiac arrest. The plasma samples were centrifuged and frozen (-70°C) immediately after collection. After the end of the study, samples were thawed once, centrifuged at 4,000 rpm for five minutes, aliquoted, and refrozen (-70°C) for later analysis. During aliquoting, the samples were kept on ice. We compared the levels of plasma NfH with the previously analysed levels of plasma NSE at the 48 hour time-point. This time-point for NSE was chosen because it had the best sensitivity/specificity for a poor prognosis in our previous Receiver Operating Characteristic (ROC) curve analysis [12]. Levels of plasma NfH were also compared with the results of SSEP-recordings approximately 72 hours after normothermia.

### NfH ELISA

All samples were coded. Plasma NfH levels were measured in duplicates with the analyst being blinded to all other information using a standard in-house ELISA [27]. Adhering to a previously proposed nomenclature the capture antibody (SMI 35 for variously phosphorylated NfH) is shown in the superscript as NfH^SMI35^. To minimize the analytical error all samples were batch analysed [28]. Batch analysis improved the analytical error (coefficient of variation) to 5.4% in the present study. The reported sensitivity of the ELISA is 0.2 ng/mL [27]. Following batch analysis the detection limit in this study was 0.001 ng/mL. Non-measurable NfH^SMI35 ^levels were reported as 0 ng/mL.

### Statistical analysis

The coded biomarker data and clinical data were electronically transferred to a third centre (Department of Neurosurgery, Copenhagen University, Denmark). Only after merging all code data the joint dataset was released to the investigators for analysis of the predefined hypotheses. Statistical analysis was performed using the SPSS software software version 15.0 (SPSS Inc., Chicago, IL, USA). Continuous data are presented as median and inter quartile range (IQR). Categorical data are given as counts and percentages. The material was dichotomized using the primary endpoints good (best CPC 1 to 2 during six months follow up), and poor neurological outcome (best CPC 3 to 4 during six months follow up). The data were not normally distributed, consequently non-parametrical statistics were used. The Mann Whitney U test was used to assess differences between the good and poor outcome groups at the different time-points. Bonferroni-corrections were used to correct for multiple comparisons. Correlations between continuous variables were assessed using Spearman correlation. Chi squared test was used to compare frequencies. ROC analysis was performed. *P *< 0.05 was considered significant.

## Results

Ninety-two patients were included and two were excluded due to all samples missing (*n *= 1) and failed analysis (*n *= 1), resulting in 90 remaining patients. For patient characteristics, see Table 1. The median time from cardiac arrest to return of spontaneous circulation was 20 minutes (IQR 14 to 30 minutes). Therapeutic hypothermia was induced at a median of 66 minutes (IQR 54 to 94 minutes) after cardiac arrest, and goal temperature was reached at a median of 210 minutes (IQR 135 to 295 minutes). Forty-eight patients (53%) had a good outcome, defined as best CPC 1 to 2 during follow up, and 42 (47%) had a poor outcome. At six months follow up, 46 patients were still alive in the good outcome group and one more in the poor outcome group resulting in a six-month survival of 52% (47 of 90). For time and cause of death and withdrawal of intensive care, see Table 2. The patients were heterogeneous with regard to their past medical history with significant co-morbidity. Fifteen of the included patients had a previously known neurological disease; stroke or intracranial bleeding for more than one year ago (*n *= 8), epilepsy (*n *= 2), posttraumatic para-paresis (*n *= 1), diabetes with polyneuropathy (*n *= 1), Guillian-Barré syndrome (*n *= 1), diffuse memory problems (*n *= 1), and placement of a ventriculoperitoneal shunt for posttraumatic hydrocephalus (*n *= 1).

**Table 1:** Patient characteristics

	Good outcome, *n *= 48	Poor outcome, *n *= 42	*P *value
Age (median, IQR)	62 (50-73)	71 (52-78)	0.062
Male sex	37 (88%)	26 (62%)	0.166
Out-of-hospital cardiac arrest	41 (85%)	36 (86%)	1.000
Initial rhythm VT/VF (*n *= 85)	35 (78%)	23 (58%)	0.001
Cardiac cause	39 (81%)	23 (55%)	0.011

*n *= 90, if not otherwise stated

Good outcome is defined as a best cerebral performance categories scale 1 to 2 during six months follow up; *P *value from Mann Whitney U test (age), Fisher's exact test for remaining comparisons.

IQR, inter quartile range; VT/VF, pulse-less ventricular tachycardia or ventricular fibrillation.

**Table 2:** Time and cause of death

	Circulatory/MOF	Neurological	Other
Number of patients	8	33	2

Time of death (d)	2 (2-32)	7 (3-17)	29 and 46

Withdrawal of intensive care	0	28	0

Time of withdrawal (d)	NA	6 (3-10)	NA

Assessment of cause of death in dying patients, including time of death, the number of patients where intensive care was withdrawn, and time of withdrawal. In patients where intensive care was withdrawn due to neurological reasons the cause of death was assessed as due to neurological reasons. Other includes premorbid disease and other interfering disease (septicaemia) unrelated to the cardiac arrest. Time is shown in days (d) as median (range). NA = not applicable; MOF = Multi-organ failure

A total of 591 samples were collected from the 90 patients (Table 3). The main reasons for missing samples were due to intra- or inter-hospital transfer (early samples), patients dying or regaining consciousness and physically leaving the ICU (late samples).

**Table 3:** Plasma NfH^SMI35 ^and neurological outcome

	Good outcome, *n *= 48				Poor Outcome, *n *= 42				
Time	n	Median	IQR	Range	n	median	IQR	Range	*P *value
Acute	29	0	0-0.25	0-3.96	28	0.05	0-0.21	0-2.47	0.586
2 h	38	0	0-0.20	0-2.35	29	0.28	0.03-0.69	0-2.83	0.002
6 h	40	0	0-0.06	0-9.21	35	0	0-0.19	0-1.35	0.569
12 h	45	0	0-0.03	0-6.96	40	0	0-0.13	0-1.21	0.172
24 h	43	0	0-0.03	0-3.96	39	0	0-0.11	0-4.82	0.035
36 h	42	0	0-0	0-0.57	34	0.05	0-0.26	0-4.14	0.000
48 h	45	0	0-0.03	0-1.20	33	0.04	0-0.15	0-0.89	0.059
72 h	40	0	0-0.02	0-1.81	31	0.01	0-0.24	0-2.23	0.024

h, hours after cardiac arrest; IQR, interquartile range; NfH, neurofilament heavy chain; *P*, Mann-Whitney U-tests without Bonferroni corrections.

**Figure 1:**
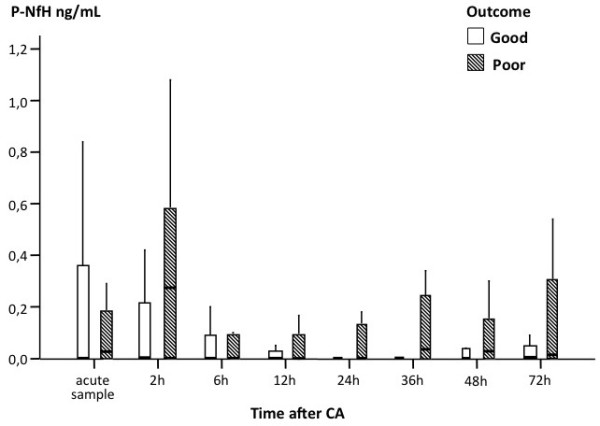
**Box-plot comparing good and poor outcome groups of patients at the different sampling times**. Boxes show the inter quartile range with median marked in the box, whiskers denote values within 1.5 times the interquartile range. Outliers are omitted. CA, cardiac arrest; NfH, neurofilament heavy chain.

**Figure 2:**
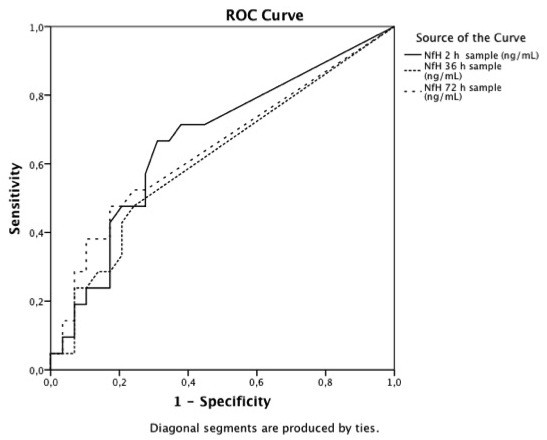
**ROC-curve showing 2, 36 and 72 hours after the cardiac arrest**. NfH, neurofilament heavy chain; ROC, receiver operating characteristic.

After Bonferroni corrections, there were significant differences between the good and the poor outcome groups at two hours (*P *= 0.016) and 36 hours (*P *< 0.005) after cardiac arrest. Plasma NfH^SMI35 ^levels were also detectable following cardiac arrest in patients with a good clinical outcome. Due to overlapping NfH^SMI35 ^values, no clinically meaningful cut-off levels separating the good and the poor outcome groups could be identified (Figure 1). The ROC-analysis showed the highest area under curve (AUC) for two hours (AUC 0.72, 95% confidence interval (CI) 0.54 to 0.90), 36 hours (AUC 0.71, 95% CI 0.52 to 0.90) and 72 hours (AUC 0.68, 95% CI 0.50 to 0.88) respectively (Figure 2). The AUC for the other measurement times were 0.51 to 0.60 also with wide CIs. Regarding six months survival, a group difference remained after Bonferroni corrections at 36 hours (*P *= 0.008) but was also significant at 72 hours (*P *= 0.04) after cardiac arrest. The plasma NfH^SMI35 ^concentration at 36 hours was selected for comparison with NSE at 48 hours to limit the number of comparisons, but no significant correlation was found (*P *= 0.068, data not shown). Plasma NfH^SMI35 ^levels at 36 hours were not significantly different between patients lacking SSEP bilaterally and patients with at least unilateral identifiable N20 peaks (data not shown).

In approximately one third of the patients the cardiac arrest was of non-cardiac cause (asphyxia/respiratory failure (*n *= 9), pulmonary embolism (*n *= 5), hanging and drowning (*n *= 3), hyperkalemia (*n *= 1), alcohol/epilepsy/acidosis (*n *= 3), and unknown (*n *= 5)). A *post hoc *analysis of the NfH values using only the patients with cardiac arrest of a cardiac cause (*n *= 62) was made. The results after Bonferroni-corrections (x8) were significant for two hours (*P *= 0.008), 24 hours (*P *= 0.048), and 36 hours (*P *< 0.005). As in the primary analysis no cut-off values were identifiable.

## Discussion

This study evaluated serial samples of NfH^SMI35 ^in plasma from patients resuscitated after cardiac arrest and treated with induced hypothermia. The main findings were that NfH^SMI35 ^was measurable in plasma from patients with poor as well as a good outcome, with significantly higher acute levels (2 and 36 hours) in the poor outcome group. The respective AUC (0.72 and 0.71) were promising but the considerable overlap of data between the groups precluded calculation of a definite cut-off value to be recommended for use in individual patients.

An ideal biomarker should be easily collected and is, in practice, often drawn from blood. Regarding biomarkers assessing neurological damage, the cerebrospinal (CSF) compartment may allow for more specific detection of central nervous system-related damage and is therefore often used [22,29]. Patients exposed to modern cardiac care, including most cardiac arrest patients, are heavily anti-coagulated and exposed to drugs affecting platelet function as well as low molecular weight heparin. This contraindicates lumbar puncture for collection of CSF due to the risk of epidural hematoma. In the ICU setting, there is instead a considerable advantage in blood sampling, allowing for serial measurements, reducing the risk of making an erroneous decision based on one single sample. Hence, although the CSF may be a more relevant compartment, assessment of biochemical neuro-markers in cardiac arrest patients is better performed in blood.

NfH is a large protein with a molecular weight of 190 to 210 kD depending on the degree of phosphorylation [18]. Following neuro-axonal injury NfH is released into the extracellular fluid from where it can be measured using ELISA [30]. From the extracellular fluid NfH diffuses into the CSF compartment. From the CSF, NfH reaches the blood via the blood CSF barrier at the lumbar level or it may diffuse directly through the cortical arachnoid villi to the blood stream [29]. The physiological wash out pattern of NfH^SMI35 ^from the CSF is not known in cardiac arrest. For reasons already discussed, it was not possible to evaluate the assessment of NfH^SMI35 ^kinetic from the CSF to the blood in this study.

Our time-course analysis showed an initial increase in plasma levels of NfH, with significant differences between the good and the poor outcome groups as early as two hours after cardiac arrest (Figure 1). This may reflect early neuronal damage [30]. From experimental data it was shown that blood NfH levels reach their peak between 30 minutes and two hours after 'cardiac arrest' [19]. This fits well with the *in vivo *human data from this study. Alternatively, NfH^SMI35 ^could be released from peripheral axons [18,19] damaged during resuscitation. The levels of plasma NfH^SMI35 ^in our study were similar to those seen within six hours after a mild stroke [23]. However, despite a uniform protein standard, one needs to be cautious when comparing these studies, because one was performed from serum and the other from plasma. The long-term temporal dynamics of blood NfH following cardiac arrest are less well known. A secondary increase of NfH^SMI35 ^occurred in the poor outcome group at later sampling times (Figure 1). We also found significant differences between NfH ^SMI35 ^levels in surviving and dying patients at 36 and 72 hours after cardiac arrest. This delayed release of NfH in this exploratory study fits with the delayed apoptotic cell death pattern described in experimental models of CA [3] and with the release pattern of the more established biomarker NSE, which peaks at 48 to 72 hours after the cardiac arrest [12]. Part of the lack of significance may relate to the fact that approximately one third of the patients had cardiac arrests of non-cardiac cause, as suggested by the *post hoc *subgroup analysis. It is also possible that complications in the post-arrest period add to development of secondary brain damage contributing to the later rise in blood levels Still, we were not able to identify any cut-off levels or predictive values to be recommended for individual patients due to a considerable data overlap. One reason may be that sampling was stopped at 72 hours after cardiac arrest and that the optimal time for NfH sampling thereby may have been missed. An increase in NfH^SMI35 ^approximately one week after subarachnoidal hemorrhage has been observed in ventricular CSF [31] and a recent study showed that NfH^SMI35 ^levels increased in serum three weeks after stroke [32]. From a practical point of view, a later maximum level of NfH ^SMI35 ^(> 1 week) is of less value for the acute prognostication and the decision on level of care. As the sensitivity of established methods for neurological prognostication, such as SSEP, is limited, biomarkers, such as serum NfH^SMI35 ^may provide complimentary information. We did not, however, find NfH^SMI35 ^levels to be related to a lost SSEP N20 potential or to the serum levels of NSE. In a previous study we found NSE to correlate well with neurological outcome [12], SSEP, diffusion-weighted MRI and also neuropathology in a fraction of the present cohort [10]. It is possible that the heterogeneous patient population and methodological limitations further discussed below, might have affected the results of the analyses. A future, larger prospective study with a prolonged sampling may allow to address these issues. Such a study should also include an appropriate control group without neurological co-morbidities to allow for better calculation of a cut-off level.

### Limitations of the study

In this study, the neurofilament analyses were performed on stored samples and the results were not available during the treatment and evaluation of patients, and have thus not influenced clinical decision-making. We encountered large variability of the plasma levels of NfH, which can be noted in the range of values in Table 3. Occasional values in one patient were high without an apparent clinical correlate and occasional patients with definite brain damage did not have measurable plasma levels of NfH. This variability needs to be taken into account when planning future studies. Long-term storing and handling of samples may have affected NfH^SMI35 ^levels and contributed to the variability.

As presented in the results section, some (*n *= 15) of the patients had neurological comorbidities. Importantly, in all of these co-morbidities, an increase of NfH^SMI35 ^levels has been described. In an experimental setting these limitations can be overcome, revealing a more meaningful temporal time course of blood NfH^SMI35 ^levels [19], but this is not realistic for clinical practice. The relevance of pre-existing neurological co-morbidity is further underlined by a *post-hoc *analysis showing significant difference in plasma NfH^SMI35 ^levels 36 hours after cardiac arrest between the patients with previous neurological disease compared with the ones without (*P *= 0.018, *n *= 76). We speculate that an already injured brain may be more vulnerable to ischemic damage following cardiac arrest but the present study was underpowered to investigate this *post-hoc *hypothesis. The presence of anti-neurofilament auto-antibodies may have masked the relevant binding epitopes for the ELISA causing false-negative results [18] but this was not investigated. Could injury to the peripheral nervous system have caused an increase of plasma NfH^SMI35 ^levels? This is a possibility and has been discussed elsewhere [29]. Particularly in patients with significant vascular co-morbidity and diabetes there is an increased likelihood for presence of coexisting peripheral neuropathy. Taken together these pre-analytical and analytical problems need to be taken into account for the interpretation of plasma NFH^SMI35 ^levels.

## Conclusions

Plasma NfH ^SMI35 ^levels were quantifiable in comatose patients after cardiac arrest. The plasma levels were significantly higher levels in the poor outcome group as compared with the good outcome group in the early phase after cardiac arrest when sedation limits the clinical assessment. Due to a considerable overlap between the groups no clinically relevant cut-off level could be proposed. It is too early to recommend the routine use of plasma NfH ^SMI35 ^in this context for clinical decision-making.

## Key messages

• NfH ^SMI35 ^is quantifiable in plasma after cardiac arrest.

• Levels are significantly higher in the poor outcome group at 2 and 36 hours after cardiac arrest.

• This exploratory study needs validation in a larger and more homogenous cohort before plasma NfH can be recommended for routine neuroprognostication.

## Abbreviations

aEEG: amplitude integrated electroencephalogram; AUC: area under curve; CI: confidence interval; CPC: cerebral performance categories scale; CSF: cerebrospinal fluid; DW-MRI: diffusion-weighted magnetic resonance imaging; ELISA: enzyme-linked immunosorbent assay; GCS: Glasgow coma scale; GFAP: glial fibrillary acidic protein; IQR: inter quartile range; NfH: neurofilament heavy chain; NfL: neurofilament light chain; NfM: neurofilament medium chain; NSE: neuronspecific enolase; ROC: receiver operating characteristic; ROSC: return of spontaneous circulation; SSEP: somatosensory evoked potential.

## Competing interests

The authors declare that they have no competing interests.

## Authors' contributions

MR and HF performed the collection of patient data, informed consents and plasma samples. TC was primarily responsible for the neurological assessment, BR combined the clinical and biochemical data sets to keep the analyst blinded to clinical outcome, AP performed the NfH analyses. HF and AP had the ideas for this study. All authors contributed in the writing process. All authors read and approved the final manuscript.
